# Studies on the genus *Psephenothrips* Reyes from China (Thysanoptera, Phlaeothripinae), with one new species

**DOI:** 10.3897/zookeys.1029.64531

**Published:** 2021-04-08

**Authors:** Li-Hong Dang, Lin-Peng Zhao, Yan-Qiao Li, Ge-Xia Qiao

**Affiliations:** 1 School of Bioscience and Engineering, Shaanxi University of Technology, Hanzhong, 723000, China Shaanxi University of Technology Hanzhong China; 2 Shaanxi Changqing National Nature Reserve, ChangqingJiayuan, No.176 DongyiHuan Road, Hanzhong, Shaanxi 723000, China Shaanxi Changqing National Nature Reserve Hanzhong China; 3 Key Laboratory of Zoological Systematics and Evolution, Institute of Zoology, Chinese Academy of Sciences, No.1 Beichen West Road, Chaoyang District, Beijing 100101, China Institute of Zoology, Chinese Academy of Sciences Beijing China; 4 College of Life Science, University of Chinese Academy of Sciences, No. 19, Yuquan Road, Shijingshan District, Beijing 100049, China University of Chinese Academy of Sciences Beijing China

**Keywords:** COI, key, *Liothrips*-lineage, new species, *Psephenothrips eriobotryae*

## Abstract

Species from China of the Asian genus *Psephenothrips* are reviewed, with *P. eriobotryae***sp. nov.** collected from the leaves of *Eriobotrya japonica* in Sichuan Province. A key to the five species known from China is provided and the COI sequence of the new species is also given.

## Introduction

Phlaeothripinae, the larger of the two subfamilies of Phlaeothripidae, is generally accepted as including three groups, Haplothripini, *Liothrips*-lineage and *Phlaeothrips*-lineage, with the last one considered to be complex and polyphyletic (Buckman et al. 2013). Of the other two, reasonably well-defined major groups, Haplothripini has been given a formal tribal name (Minaei and Mound 2008), but the *Liothrips*-lineage of leaf-feeding species remains confused and difficult to interpret. The major reason is that about half of the genera are monobasic, with many poorly-defined genera and no clear suprageneric classification (Stannard 1957; Mound and Marullo 1996; Buckman et al. 2013; Dang et al. 2014). There is a lack of identification systems worldwide to members of the *Liothrips*-lineage and, for China, a serious lack of field studies of species and genera of the *Liothrips*-lineage. Dang et al. (2014) provided a key to 100 genera from southeast Asia and China, including 34 genera associated with *Liothrips*. More recently, Wang and Lin (2020) published keys to 11 genera and 28 species of *Liothrips*-lineage from Taiwan. However, there is a lack of information about the *Liothrips* fauna of mainland China and preparation of suitable identification systems will require a great deal of work studying slide-mounted specimens and establishing host associations through extensive field work.

As part of attempted studies on the *Liothrips*-lineage from China, the Asian genus *Psephenothrips* is reviewed here. This is one of the poorly-defined genera associated with *Liothrips*, with which it shares the following characteristics: antennal segment III with one outer sense cone, segment IV with 1+2 sense cones and prosternal basantra absent. However, species of this genus differ in having the maxillary stylets long and close together in the middle of the head, the fore tarsal tooth absent in both sexes and the male sternite VIII without a pore plate (Okajima 2006; Tyagi and Kumar 2012). Worldwide, there are seven species listed in this genus (ThripsWiki 2021), of which the type species *P. strasseni* was described from Philippines, *P. leptoceras*, *P. cinnamomi* and *P. machili* from Japan and *P. moundi* from India (Reyes 1994; Okajima 2006; Tyagi and Kumar 2012). Recently, Wang and Lin (2020) reported four species in this genus from Taiwan, of which *P. baiheensis* and *P. cymbidas* were new species. Presumably, these species all feed on green leaves, although *P. leptoceras* and *P. baiheensis* were collected from dead branches and wood. The new species *P. eriobotryae* sp.nov. is described here from Sichuan Province and *P. leptoceras* from Yunnan Province is newly recorded from the Chinese mainland. A key to five species from China is provided and the COI sequence of the new species is also given.

## Methods

The descriptions, photomicrograph images and drawings were produced from slide-mounted specimens using an Olympus BX53 microscope with a drawing tube. The abbreviations used for the pronotal setae are as follows: am – anteromarginal, aa – anteroangular, ml – mid-lateral, epim – epimeral, pa – posteroangular. The unit of measurements is the micrometre. All specimens studied here are deposited in the School of Bioscience and Engineering, Shaanxi University of Technology (**SUT**), Hanzhong, China, with some specimens in the National Zoological Museum of China (**NZMC**), Institute of Zoology, Chinese Academy of Sciences, Beijing, China.

## Taxonomy

### Psephenothrips

Taxon classificationAnimaliaThysanopteraPhlaeothripidae

Reyes

83E85954-EAF1-59B5-9838-6387848C3872

Psephenothrips Reyes, 1994: 478. Type species: Psephenothrips strasseni Reyes.

#### Diagnosis.

Head as long as broad or longer than broad; postocular setae well developed, shorter or as long as eyes. Antenna eight-segmented, segment III with 0+1 sense cone, IV with 1+2 sense cones. Maxillary stylets long, reaching eyes and close together in the middle of head and maxillary bridge absent. Prothorax usually with five pairs of setae, sometimes am minute; notopleural sutures complete; basantra absent. Mesopresternum boat-shaped or divided into two parts; metathoracic sternopleural sutures present or absent. Fore tarsus without tooth in both sexes. Fore wing parallel-sided, with numerous duplicated cilia. Pelta broadly triangular; abdominal tergites II–VII with two pairs of sigmoid wing-retaining setae; tergite IX with S1 and S2 setae shorter or as long as tube. Male without a pore plate on abdominal sternite VIII.

#### Comments.

The species of *Psephenothrips* are similar to *Liothrips*, but they are distinguished by the long maxillary stylets that are close together in the middle of the head and abdominal segment VIII without a pore plate in the male. This genus is also closely related to *Eurhynchothrips* in body shape as well most characters including those above (Mound 1968). In this study, these two genera could not be separated from each other. However, according to the generic diagnosis (Mound 1968; Dang et al. 2014), *Psephenothrips* can be distinguished from *Eurhynchothrips* only by having no stout maxillary bridge and that is the reason this new species, *P. eriobotryae* sp.nov., is considered to be a species of *Psephenothrips*. They both are poorly-defined genera in the *Liothrips*-lineage and the relationship between them is extremely confused and requires further considerable study.

### Key to *Psephenothrips* species from China

^*^from descriptions; Okajima 2006; Wang and Lin 2020.

**Table:** 

1	Four pronotal major setae well-developed, am setae minute	***eriobotryae* sp.nov.**
–	Five pronotal major setae well-developed including am setae	**2**
2	Metathoracic sternopleural sutures absent or vestigial	**3**
–	Metathoracic sternopleural sutures distinct	**4**
3	Antennal segment VIII about as long as VII	***leptocerus***
–	Antennal segment VIII shorter than VII	machili ^*^
4	Mesopresternum boat-shaped with a sharp median protruding	baiheensis ^*^
–	Mesopresternum narrowly connected or separated into 2 parts	cymbidas ^*^

### Psephenothrips baiheensis

Taxon classificationAnimaliaThysanopteraPhlaeothripidae

Wang & Lin

7A484A35-9F99-595D-96EF-6E1B2078B3BB

Psephenothrips baiheensis Wang & Lin, 2020: 371.

#### Comments.

Described recently from Taiwan, this species was based on three females collected from dead wood. However, as it shares the characteristics of the *Liothrips*-lineage, it presumably feeds on green leaves. In this study, no specimen was examined, but antennal segments VI–VIII were described as uniformly dark brown and this distinguishes the species within the genus *Psephenothrips*.

### Psephenothrips cymbidas

Taxon classificationAnimaliaThysanopteraPhlaeothripidae

Wang & Lin

896D4F76-431B-5014-A001-58D28AB007AE

Psephenothrips cymbidas Wang & Lin, 2020: 371.

#### Comments.

As with *P. baiheensis*, this species was described from Taiwan and was based on six females and three males from *Cymbidium*. We did not see any specimens of *P. cymbidas* and the original description “mid tibiae greyish brown and fore wings pale brown” is not strong evidence for distinguishing the species from *P. strasseni* and other *Psephenothrips* species. The mesopresternum, divided into two parts that may be narrowly connected, could be an obvious distinguishing characteristic, but its form needs to be verified in further study.

### Psephenothrips eriobotryae

Taxon classificationAnimaliaThysanopteraPhlaeothripidae

Dang & Qiao sp.nov.

A4F88D62-2F48-5727-BB9B-97A3EEF970A1

http://zoobank.org/7DCC1A9C-8306-4E14-9ACA-0918D510C950

Figs 1–7 , 8–15

#### Material examined.

***Holotype*.** ♀ (SUT), China, Sichuan Province, Guangyuan City, Fenghuangshan Park, on the leaves of loquat tree (*Eriobotrya japonica* (Thunb.) Lindl, 07.viii.2018, L.H. Dang, Y. Hu & D.L. Xie. ***Paratypes*.** 3♂, with the same data as holotype.

#### Diagnosis.

**Figures 1–7.**
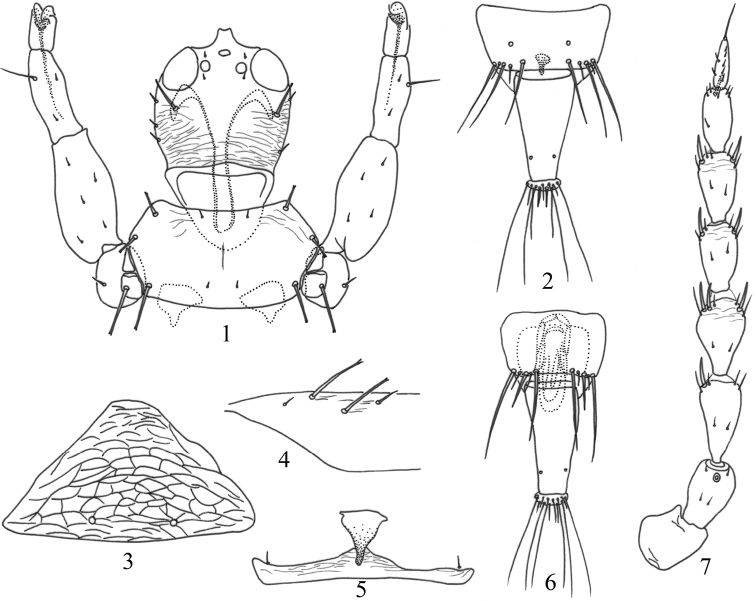
*Psephenothrips eriobotryae* sp. nov. **1** head, pronotum and fore legs **2** tergites I, female **3** pelta **4** base of forewing **5** mesopresternum **6** tergites IX–X, male **7** antenna.

**Figures 8–15.**
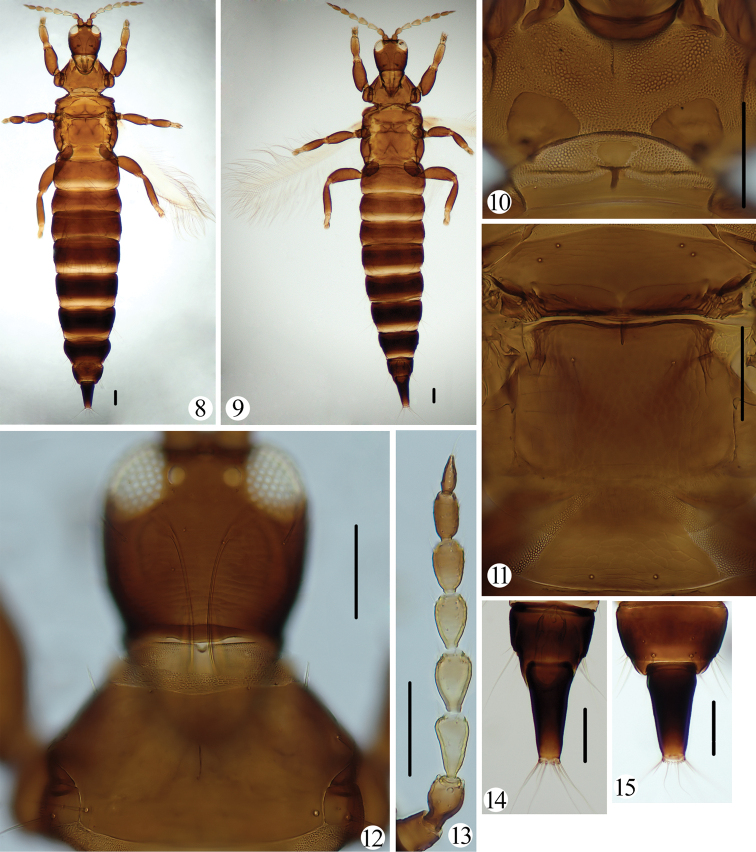
*Psephenothrips eriobotryae* sp. nov. **8** adult, female **9** adult, male **10** prosternum and mesopresternum **11** meso-, metanotum and pelta **12** head and pronotum **13** antenna **14** tergites IX–X, male **15** tergites IX–X, female. Scale bars: 100 μm.

Body uniform brown (Fig. 8); head as long as wide (Figs 1, 8), postocular setae shorter than eyes, very weakly expanded at apex (Fig. 1); maxillary stylets retracted into eyes, little wide apart (Figs 1, 8); pronotal am minute, other four pairs of major setae well-developed, weakly expanded at apex (Fig. 1); mesopresternum boat-shaped with a protrusion (Figs 5, 10); metathoracic sternopleural sutures absent; fore wing sub-basal setae S1 about as long as S2, expanded at apex, S3 small, pointed (Fig. 4); female abdominal tergite IX setae S1 and S2 shorter than tube (Figs 2, 15); male abdominal sternite without a pore plate, S2 setae of tergite IX short and stout (Figs 6, 14).

#### Description.

***Holotype*. *Female macroptera*.** Body brown (Fig. 8). Antennal segments I–II and VII–VIII brown, III yellow, IV–V yellow, but apically brownish, VI brown with basal part yellow (Fig. 13); fore tibiae yellowish-brown with brown at basal 1/3, all tarsi yellow, rest of legs brown (Fig. 8). All major setae yellowish-brown.

***Head*.** Head as long as wide (Figs 1, 8); dorsal surface weakly sculptured with transverse striae, ocellar region smooth; postocular setae shorter than eyes, very weakly expanded at apex (Fig. 1); cheeks gradually narrowed towards base, with a few stout setae; mouth-cone rounded, maxillary stylets retracted into eyes, little wide apart (Figs 1, 8). Antennae 8-segmented, VIII weakly constricted at base, slightly shorter than VII, sense cones stout, III with 0+1, VI with 1+2, V–VI with 1+1, respectively (Fig. 7).

***Thorax*.** Pronotum almost smooth, notopleural sutures complete (Figs 1, 12), am minute, other four pairs of major setae well-developed, weakly expanded at apex, epim about as long as pa (Fig. 1); metanotum weakly sculptured with reticulation, with pointed setae (Fig. 11); mesopresternum boat shaped with a protruding (Figs 5, 10), metathoracic sternopleural sutures absent. All legs normal, without fore tarsal tooth (Fig. 1); fore wing parallel sided (Fig. 8), with 8–14 duplicated cilia, sub-basal wing setae arranged in a triangle, S1 about as long as S2, expanded at apex, S3 small, pointed (Fig. 4).

***Abdomen*.** Pelta round triangled and reticulate (Figs 3, 11); abdominal tergites II–VII with two pairs of wing-retaining setae; tergite IX setae S1 and S2 shorter than tube, nearly pointed or narrowly blunt at apex (Figs 2, 15); tube about 2.0 times as long as basal width, anal setae slightly shorter than tube.

***Measurements*** (holotype female in microns). Body length 2545. Head length 215, width across eyes 215; eye length 83, width 65; postocular setae length 38. Antennae length 450, segments I–VIII lengths 46, 58, 65, 60, 55, 55, 44 and 41. Pronotum length 163, width 315, length of pronotal setae, am 5, aa 30, ml 25, epim 65, pa 65. Fore wing length 895, wing sub-basal S1–S3, 40, 35 and 15. Pelta length 110, width 200; tergite IX posteromarginal setae S1–S3, 105, 85 and 102; tube length 165, basal width 88, width in the middle 68, at apex 38; anal setae length 160.

***Male macroptera*.** Very similar to female (Fig. 9), fore legs without fore tarsal tooth (Fig. 9); abdominal sternite without a pore plate, S2 setae of tergite IX short and stout (Figs 6, 14).

***Measurements*** (paratype male in microns). Body length 2190. Head length 195, width across eyes 203; eye length 75, width 65; postocular setae length 60. Antennae length 460, segments I–VIII length 42, 60, 65, 62, 57, 57, 55 and 45. Pronotum length 150, width 370, length of pronotal setae, am 10, aa 38, ml 40, epim 75, pa 56. Fore wing length 860, wing sub-basal S1–S3, 38, 30 and 20. Pelta length 95, width 175; tergite IX posteromarginal setae S1–S3, 120, 45 and 155; tube length 185, basal width 90, width in the middle 65, at apex 38; anal setae length 155.

#### COI sequence.

It includes 1492 bp with the GenBank number MW567215.

#### Etymology.

This species name is composed of one Latin word, *eriobotryae*, based on its host plant.

**Figures 16–25.**
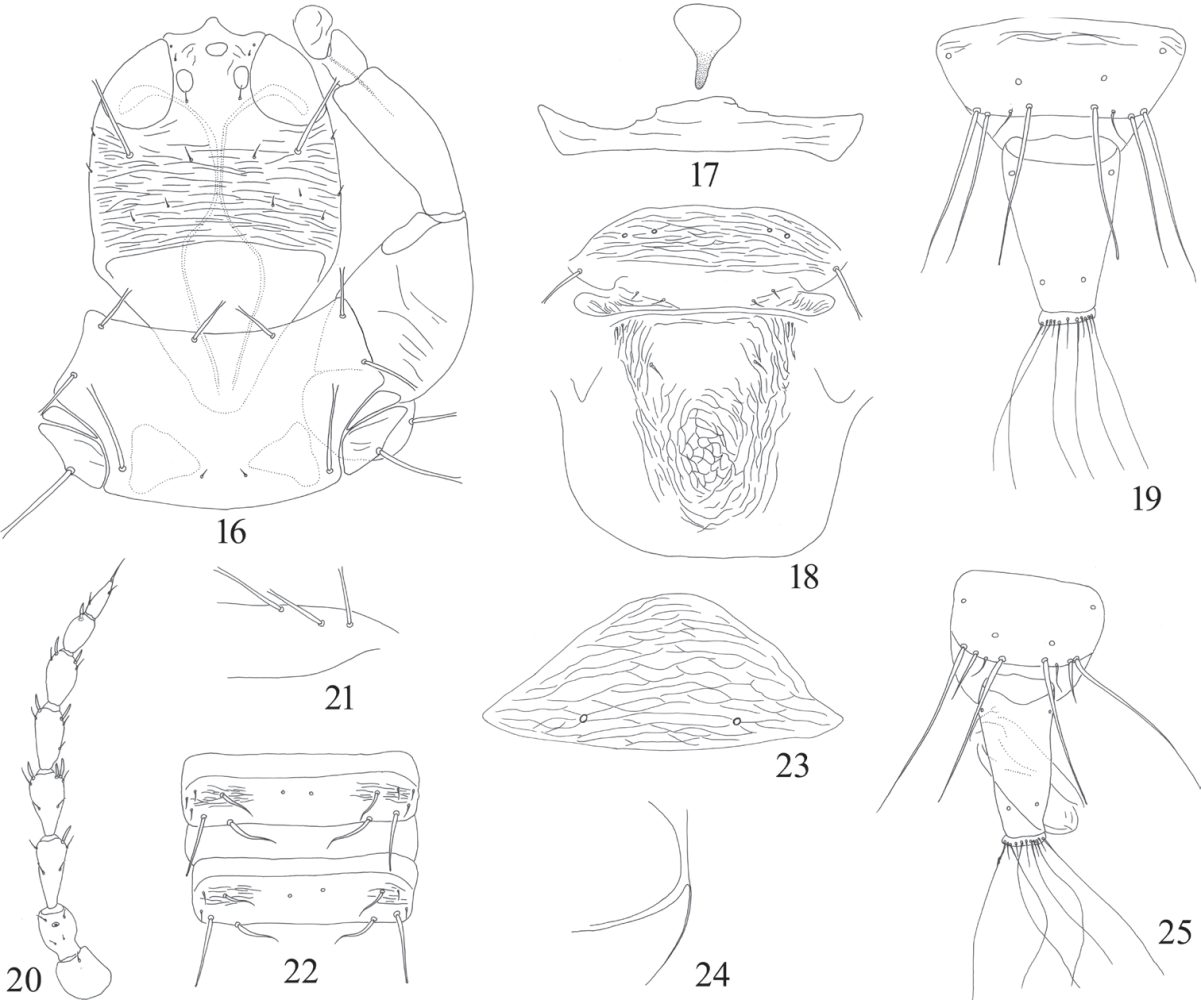
*Psephenothrips leptoceras***16** head, pronotum and fore leg **17** mesopresternum **18** meso- and metanotum **19** tergites IX–X, female **20** antenna **21** base of forewing **22** tergites V–VI **23** pelta **24** metathoracic sternopleural suture, left **25** tergites IX–X, male.

**Comments.** This new species can be distinguished from the other members of *Psephenothrips* by having pronotal am minute. It is similar to *P. leptoceras* in body shape and colour, but differs in having postocular setae shorter than eyes (Figs 1, 8), sub-basal setae S3 small and pointed at apex (Fig. 4), and posteromarginal setae of tergite IX much shorter than tube (Figs 2, 15). In *P. leptoceras*, the postocular setae are about as long as eyes (Fig. 16), sub-basal setae S3 are as long as S2 and expanded at apex (Fig. 21), and posteromarginal setae of tergite IX are slightly shorter than tube (Fig. 19).

### Psephenothrips leptoceras

Taxon classificationAnimaliaThysanopteraPhlaeothripidae

Okajima

2B384F65-C5A3-5485-BABD-E573A0416E39

Figs 16–25

Psephenothrips leptoceras Okajima, 2006: 554.

#### Comments.

Described from Japan on dead branches, this species was recorded from Taiwan on *Rhus semialata* by Wang and Lin (2020). It is here recorded from the Chinese mainland in Yunnan Province for the first time, based on five females and one male from a mango tree. Although the original description stated this species was collected from dead branches, it could be feeding on green leaves.

### Psephenothrips machili

Taxon classificationAnimaliaThysanopteraPhlaeothripidae

(Moulton)

DAA9109B-9CD1-55E6-83A1-7329759024FF

Rhynchothrips machili Moulton, 1928: 313.

#### Comments.

Described from Taiwan by Moulton (1928), this species was transferred by Okajima (2006) to the genus *Psephenothrips* after checking the types and many specimens from Japan. Subsequently, *Liothrips machilus*Ananthakrishnan and Varadarasan (1978) from India was synonymised with *P. machili* by Tyagi and Kumar (2012), based on all paratypes and three females. These specimens were all collected from leaves of *Machilus* sp., but no specimen was examined in the present study. According to the re-description by Okajima (2006), this species is similar to *P. eriobotryae* sp.nov., but can be distinguished by the well-developed pronotal am setae and sub-basal wing setae S3 developed and expanded at the apex. Moreover, they feed on different plants.

## Supplementary Material

XML Treatment for Psephenothrips

XML Treatment for Psephenothrips baiheensis

XML Treatment for Psephenothrips cymbidas

XML Treatment for Psephenothrips eriobotryae

XML Treatment for Psephenothrips leptoceras

XML Treatment for Psephenothrips machili
